# Using alternative programming algorithms and techniques for optimizing performance of the Casemix software

**DOI:** 10.1186/1472-6963-12-S1-O10

**Published:** 2012-11-21

**Authors:** H Reeza, A Zafar, AJ Hamzah, Syed Aljunid

**Affiliations:** 1 International Training Centre for Casemix and Clinical Coding (ITCC), UKM Medical Centre, Kuala Lumpur, Malaysia; 2 United Nations University-International Institute for Global Health, Kuala Lumpur, Malaysia

## Introduction

Casemix system is a patient classification system that classifies patients into predefined groups based on the patient level data. Because of the immense variation of the individual patient level data it is very tedious to do this Casemix group allocation manually, instead Casemix classification software with a predefined logic is used. UNU-IIGH has developed Casemix logic, and an application for easy usage by end users. As part of continuous quality improvement, it was to upgrade the Casemix software for improved data processing.

## Objective

To create an appropriate algorithm and utilize Java programming technique for optimizing Casemix software.

## Methodology

Casemix logic requires lots of data input in order to classify for each of its class. To begin with, the current Casemix logic algorithm will be broken down and analyzed. Due to multiple matching process, the system should be refined and recreated to an efficient algorithm such as binary search method to replace the current linear search method which is slow in searching, matching method and database access. The developed prototype will be tested for the efficient data processing using a 1 Million patient and the performance compared with the earlier version. The efficiency test is expected show the increased performance of new alternative programming algorithm and techniques approach for optimizing the system.

## Results

The current system in use is using linear search method which has time complexity of O(n) to do the matching process. By implementing binary search method, in the proposed solution, the time complexity is O (log n) which is proven to be faster than linear search method. The new programming algorithm and techniques used in the new prototype Casemix software are expected to increase the speed of matching process and accessing database. It is estimated that it will increase the search process 15% quicker than previous Casemix software. This enhanced performance of the newly developed prototype Casemix software will be used in future development.

